# Proposal of initial and maintenance dosing regimens with linezolid for renal impairment patients

**DOI:** 10.1186/s40360-021-00479-w

**Published:** 2021-03-04

**Authors:** Hitoshi Kawasuji, Yasuhiro Tsuji, Chika Ogami, Kou Kimoto, Akitoshi Ueno, Yuki Miyajima, Koyomi Kawago, Ippei Sakamaki, Yoshihiro Yamamoto

**Affiliations:** 1 Department of Clinical Infectious Diseases, Toyama University Graduate School of Medicine and Pharmaceutical Sciences, 2630 Sugitani, Toyama, 930-0194 Japan; 2 Center for Pharmacist Education, School of Pharmacy, Nihon University, 7-7-1 Narashinodai, Chiba, 274-8555 Japan; 3 Department of Medical Pharmaceutics, Faculty of Pharmaceutical Sciences, University of Toyama, 2630 Sugitani, Toyama, 930-0194 Japan

**Keywords:** Linezolid, Renal impairment, Thrombocytopenia, Therapeutic drug monitoring, Dosing regimen

## Abstract

**Background:**

Linezolid is administered as a fixed dose to all patients despite evidence of overexposure and thrombocytopenia in renal impairment. The aims of this study were to evaluate the risk of thrombocytopenia and the utility of therapeutic drug monitoring (TDM), and to propose alternate dosing regimens in patients with renal impairment.

**Methods:**

We retrospectively reviewed patients ≥13 years old for whom serum linezolid trough concentration (*C*_min_) was measured during linezolid treatment. Patients with episodes of infection were divided into groups by presence of renal impairment (RI group) or absence of renal impairment (non-RI group), and by use of *C*_min_-based TDM (TDM group) or not (non-TDM group) during linezolid treatment.

**Results:**

In the 108 patients examined by multivariable analyses, renal impairment was independently associated with increased risk of thrombocytopenia (OR 3.17, 95%CI 1.10–9.12) and higher *C*_min_. Analysis of the utility of TDM in the RI group showed that clinical failure rate was significantly lower in the TDM subgroup than in the non-TDM subgroup. Furthermore, in the RI group, dosage adjustments were needed in 90.5% of the TDM subgroup. All episodes administered a reduced dose of 300 mg every 12 h in the RI group showed *C*_min_ ≥ 2.0 mg/L. Additional analysis of 53 episodes in which *C*_min_ was measured within 48 h after starting administration showed that the initial standard dose for 2 days was sufficient to rapidly reach an effective therapeutic concentration in the RI group.

**Conclusions:**

Empirical dose reduction to 300 mg every 12 h after administration of the initial fixed dose for 2 days and *C*_min_-based TDM may improve safety outcomes while maintaining appropriate efficacy among patients with renal impairment.

**Supplementary Information:**

The online version contains supplementary material available at 10.1186/s40360-021-00479-w.

## Background

Linezolid is the first synthetic oxazolidinone agent that is used in the treatment of multi-drug resistant pathogens, such as methicillin-resistant *Staphylococcus aureus* (MRSA), methicillin-resistant coagulase-negative staphylococci (MR-CoNS), vancomycin-resistant Enterococci, and *Mycobacterium tuberculosis* [1, 2]. Thrombocytopenia is exposure-dependent adverse effects of linezolid treatment and sometimes leads to discontinuation, even in the short periods [3]. An exposure-response relationship has been clarified for thrombocytopenia and previous studies showed that linezolid trough concentration (*C*_min_) values above 7–8 mg/L have consistently been associated with an increased risk of thrombocytopenia [4–9].

Approximately 30–40% of the administered linezolid is excreted unchanged via the urine, and kidney function is thus a significant source of interpatient variability in linezolid clearance (CL) [10, 11]. A recent study showed that patients with impaired renal function receiving standard linezolid doses more frequently experienced thrombocytopenia [9]. Renal impairment has been identified as a significant risk factor for increased linezolid *C*_min_ in real-world clinical studies [12, 13]. However, linezolid is currently administered as a fixed dose of 600 mg every 12 h to all patients despite evidence of overexposure and thrombocytopenia in renal impairment [14]. Accordingly, therapeutic drug monitoring (TDM) and dose modification have been proposed by some authors to improve the safe and effective use of linezolid, especially in the population with renal impairment [4, 6, 9, 15].

Although linezolid overexposure has been reported to be related to several factors including renal impairment [9, 13, 15], drug-drug interactions [16], and illness severity [17], previous studies have suggested that a reduced dose of 300 mg every 12 h is better suited to patients with creatinine clearance (CL_CR_) < 30 mL/min or estimated glomerular filtration rate (eGFR) < 60 mL/min/1.73 m^2^, based on Monte Carlo simulations for sufficient efficacy and safety [9, 18]. However, real-world data from clinical practice to support this recommendation have remained lacking.

The aims of the present study were threefold: 1) to evaluate the relationships between renal impairment, thrombocytopenia and linezolid overexposure; 2) to evaluate whether TDM and TDM-guided dose modification could help prevent and/or recover from linezolid-induced myelosuppression, and prevent treatment failure with good outcome; and 3) to propose alternate initial and maintenance dosing regimens for patients with impaired renal function using actual measurement data from clinical practice.

## Methods

### Study design

We conducted a monocentric, retrospective, observational study from April 2013 to December 2019 among patients ≥13 years old who were treated with linezolid film-coated tablets and/or injections (Zyvox®; Pfizer, Tokyo, Japan) because of suspected or documented Gram-positive bacterial infections at Toyama University Hospital. Patients with at least one linezolid serum *C*_min_ measured under steady-state conditions, at least 72 h after linezolid initiation or dose modification, during linezolid treatment were eligible for inclusion. Patients receiving renal replacement therapies including hemodialysis and continuous renal replacement therapy, and patients who were administered linezolid for tuberculosis or nontuberculous mycobacterial infections were excluded. Recurrent infection within the same patient was considered a distinct episode only if it occurred more than 1 week after the initial episode and once antimicrobial therapy had been completed. CL_CR_ was estimated using the Cockcroft-Gault formula (CL_CRC-G_) and renal impairment was defined as a CL_CRC-G_ ≤ 60 mL/min at baseline. Combination antimicrobial therapy was applied whenever clinically needed.

In the present study, linezolid was designed to be started with a fixed dose to all patients, but the initial dose was finally determined at the discretion of the attending physician. Linezolid TDM was performed via infectious disease (ID) consultation upon the request of attending physicians and the results were properly reported to the physicians responsible for the patient. *C*_min_ was measured using peripheral venous blood samples collected as clinical practice, just before the next administration after starting linezolid therapy. The times of the intravenous infusions or oral administrations and blood collections were carefully checked, and samples deemed inappropriate were excluded from the analysis. All serum samples obtained were stored at − 80 °C until linezolid trough measurement. *C*_min_ values were suitably measured, especially when ID physicians and/or attending physicians decided it necessary by reference to the course of platelet counts or *C*_min_ values, until the end of treatment. When linezolid *C*_min_ > 10 mg/L and thrombocytopenia occurred in the patient, linezolid dose adjustment was recommended by ID physicians, focused on controlling linezolid *C*_min_ within the optimal range of 2–8 mg/L [4, 6, 12]. TDM-based dose adjustments were performed finally at the discretion of the attending physician. Drug dosages were scaled linearly, with a minimum dose modification of 300 mg for the oral-route tablet.

### Method of quantification

Steady-state serum *C*_min_ was defined as the total concentration just before administration of linezolid at ≥72 h after linezolid initiation or dose modification. Serum concentrations of linezolid were analyzed by means of a validated HPLC analysis method, as previously described [15]. The intra- and inter-day coefficients of variation were always < 5% and the lower limit of detection was 0.1 mg/L. If multiple steady-state *C*_min_ values at the same dosage were measured in one episode, the mean value of all measurements from that episode was used for statistical analyses.

### Analysis strategy

In the present study, episodes were divided into two subgroups, on the basis of the presence of renal impairment (renal impairment group; RI group) or absence of renal impairment (non-RI group). Patients were also divided into those for whom *C*_min_-based TDM was used for dosage adjustment during linezolid treatment (TDM group) or in whom linezolid *C*_min_ values were measured and assessed only after the end of linezolid treatment, not during treatment (non-TDM group). Among most episodes in non-TDM group, linezolid *C*_min_ values could not be measured during linezolid treatment due to delay of the requests for ID consultation from attending physician and/or difficult to secure sufficient time for immediate measurements under the condition of limited human resources capable of measure linezolid concentrations.

### Data collection

For each episode, the following data were retrieved from medical charts and written ID consultations: demographics, type of infection, isolated microorganisms, treatment duration, concomitant medications, linezolid dosage and serum *C*_min_ at each instance of TDM, number of all instances of TDM, number of instances of TDM under steady-state conditions, whether TDM for dosage adjustment was performed during linezolid treatment and whether TDM-guided dosage adjustments were performed. Hematological and serum chemistry analyses performed on each day during treatment were retrieved and compared over time.

### Clinical outcome

Episodes were defined as recovered if no clinical, biological and/or radiological evidence of infection was apparent at the end of treatment [4]. Failure was defined as any discontinuation of linezolid therapy before the end of treatment, either because of toxicity or because of persistence of infection [4]. Thirty-day reinfection was defined as infection caused by the same strain at the same infection site within 30 days after end of antimicrobial treatment.

### Safety and tolerability outcome

Thrombocytopenia was defined as platelet count < 112.5 × 10^3^/μL (75% lower limit of normal) at any time during treatment for episodes with platelet count at or above the lower limit of normal (≥ 150 × 10^3^/μL) at baseline before administration, and 25% reduction from the baseline value for episodes with low platelet counts at baseline (75–149 × 10^3^/μL) [9, 19]. Severe thrombocytopenia was defined as platelet count < 75 × 10^3^/μL for episodes with a normal baseline and platelet count < 50 × 10^3^/μL for those with low baseline platelets, respectively [9, 19].

Recovery from thrombocytopenia was defined as the return and maintenance of platelet count to > 112.5 × 10^3^/μL during therapy for episodes with platelet count at or above the lower limit of normal (≥ 150 × 10^3^/μL) at baseline, or values > 75% of baseline values with low platelet count at baseline (75–149 × 10^3^/μL), after experiencing thrombocytopenia [4]. Time to the development of thrombocytopenia was also recorded.

### Statistical analysis

The Kolmogorov-Smirnov test was used to assess the normality of data. Descriptive data are expressed as mean ± standard deviation or median with IQR, and continuous variables were compared using the Mann-Whitney test. Categorical variables were compared using the χ^2^ test with Yates’s correction or Fisher’s exact test as necessary. In all analyses, we preliminarily confirmed the affect of multicollinearity of the covariates used in the statistical analysis. Univariate logistic regression analysis was used to investigate variables potentially associated with the occurrence of thrombocytopenia. Multivariate logistic regression analyses were performed with all the independent variables showing *P* ≤ 0.10 on univariate analysis as well as with the main variable of renal impairment and variables deemed either clinically relevant or supported in the medical literature. Similarly, uni- and multivariate linear regression analyses were used to identify independent predictors of higher *C*_min_ at the fixed dose. A value of *P* ≤ 0.05 was considered statistically significant. All statistical analysis and plotting were performed using JMP Pro version 14.2.0 software (SAS Institute, Cary, NC).

## Results

### Toxicity and linezolid exposure

**Fig. 1:**
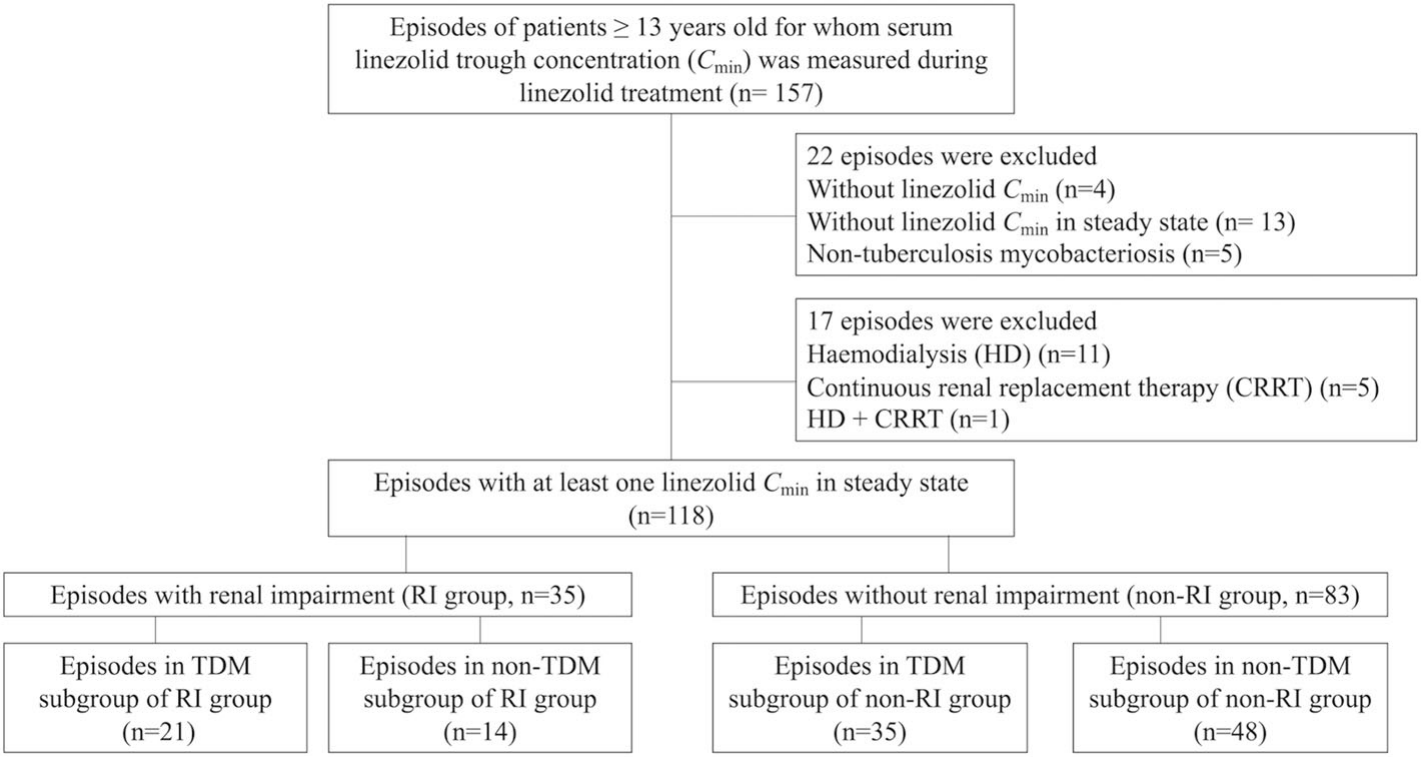
Study flow. Abbreviations: *C*_min_, trough concentration; HD, hemodialysis; CRRT, continuous renal replacement therapy; TDM, therapeutic drug monitoring; RI, renal impairment

Figure 1 depicts the study flow chart. A total of 118 episodes in 108 patients were included, comprising 35 episodes in 33 patients with renal impairment (RI group) and 83 episodes in 75 patients without renal impairment (non-RI group). All episodes except for six were initially administered as a fixed dose. The remaining 6 episodes were initially reduced to 600 mg per day because of lower body weight (≤ 45 kg) or elderly (≥ 88 years old) which were determined at the discretion of the attending physician. Demographics and clinical baseline characteristics stratified by CL_CR_ calculated using the Cockcroft-Gault formula (CL_CRC-G_) are summarized in Table 1. Episodes mainly occurred in males (64.4%) with a median (range) age of 71 years (17–95 years) and a median weight of 57.1 kg (30.4–113.0 kg). The main indications for linezolid therapy were skin and soft tissue infections and surgical site infections followed by bacteremia, bone and joint infections, and respiratory tract infections. Skin and soft tissue infections and surgical site infections were more common in the non-RI group, and bacteremia was significantly more common in the RI group. MRSA and MR-CoNS were the most frequent bacterial isolates. Therapy was empirical in 14.4% of episodes and combination antimicrobial therapy was prescribed in 54.2% of episodes.

**Table 1:** Characteristics of episodes with or without renal impairment (RI group or non-RI group, respectively)

	All, 118 episodes in 108 patients	RI group, 35 episodes (29.7%) in 33 patients	Non-RI group, 83 episodes (70.3%) in 75 patients	*P*-value
**Demographics**				
Age (years), median (IQR)	71 (58.5–78)	78 (72–82)	67 (47–74)	< 0.0001
Sex (male/female), (%/%)	76/42 (64.4/35.6)	22/13 (62.9/37.1)	54/29 (65.1/34.9)	0.84
Height (m), median (IQR)	1.61 (1.53–1.67)	1.56 (1.45–1.63)	1.64 (1.56–1.70)	0.0091
Body weight (kg), median (IQR)	57.1 (48.0–64.2)	49.4 (45.0–60.3)	59.3 (52.2–65.4)	0.0018
Body mass index (kg/m^2^), median (IQR)	22.2 (20.1–23.7)	21.0 (18.9–22.9)	22.4 (20.6–24.6)	0.017
**Laboratory, median (IQR)**				
Serum creatinine (mg/dL)	0.65 (0.50–1.00)	1.20 (0.79–1.49)	0.57 (0.48–0.74)	< 0.0001
CL_CRC-G_	76.0 (49.2–105.4)	36.2 (26.9–49.4)	93.7 (72.0–118.7)	< 0.0001
Total bilirubin (mg/dL)	0.4 (0.3–0.7)	0.4 (0.3–0.8)	0.4 (0.3–0.6)	0.85
**Baseline hematological parameters**				
Hemoglobin concentration (g/dL)	9.8 (8.5–11.6)	8.9 (8.3–10.2)	10.1 (8.7–11.9)	0.0081
Platelet count (×10^3^/μL), median (IQR)	243 (177–319)	208 (151–284)	255 (181–247)	0.062
Low platelet count at baseline < 150 × 10^3^/μL, n (%)	23 (19.5)	8 (22.9)	15 (18.1)	0.61
Episodes with platelet transfusion during therapy, n (%)	8 (6.8)	4 (11.4)	4 (4.8)	0.23
Episodes with DIC, n (%)	15 (12.7)	5 (14.3)	10 (12.1)	0.77
**Main reason for linezolid**				
**Type of infection, n (%)**				
Skin and soft tissue infections, and surgical site infections	47 (39.8)	9 (25.7)	38 (45.8)	0.063
Bacteremia	36 (30.5)	18 (51.4)	18 (21.7)	0.0021
Bone and joint infections	31 (26.3)	12 (34.3)	19 (22.9)	0.25
Respiratory tract infections	26 (22.0)	8 (22.86)	18 (21.69)	1.00
Intra-abdominal infections	8 (6.8)	4 (11.4)	4 (4.8)	0.23
Mediastinitis	7 (5.9)	2 (5.7)	5 (6.0)	1.00
Central nerve system infections	5 (4.2)	1 (2.9)	4 (4.8)	1.00
Endocarditis	4 (3.4)	2 (5.7)	2 (2.4)	0.58
Urinary tract infections	4 (3.4)	3 (8.6)	1 (1.2)	0.078
Unknown	6 (5.1)	1 (2.9)	5 (6.0)	0.67
**Microbiological isolate, n (%)**				
MRSA	63 (53.4)	18 (51.4)	45 (54.2)	0.84
MR-CoNS	25 (21.2)	8 (22.9)	17 (20.5)	0.81
Enterococci	7 (5.9)	3 (8.6)	4 (4.8)	0.42
*Enterococcus faecalis*	2 (1.7)	1 (2.9)	1 (1.2)	0.51
*Enterococcus faecium*	5 (4.2)	2 (5.7)	3 (3.6)	0.63
*Corynebacterium* species	6 (5.1)	4 (11.4)	2 (2.4)	0.063
*Bacillus cereus*	3 (2.5)	2 (5.7)	1 (1.2)	0.21
Other	8 (6.8)	4 (11.4)	4 (4.8)	0.23
No isolate, Unknown	11 (9.3)	2 (5.7)	9 (10.8)	0.50
**Linezolid dosage and exposure**				
Empirical/target therapy, n/n (%/%)	17/101 (14.4/85.6)	2/33 (5.7/94.3)	15/68 (18.1/81.9)	0.093
Dose (mg/kg/day), median (IQR)	20.7 (17.8–24.2)	23.6 (18.5–26.7)	20.0 (17.3–22.3)	0.017
Mean *C*_min_ of fixed doses at steady state (mg/L), mean ± SD	17.3 ± 10.5	25.6 ± 10.4	14.1 ± 8.8	< 0.0001
Number of all TDM instances, median (IQR)	6 (3–8)	6 (4–11)	6 (2–8)	0.33
Number of TDM instances under steady-state conditions, median (IQR)	3 (2–6)	3 (2–6)	3 (2–6)	0.47
Episodes with TDM assessment performed during linezolid treatment, until end of treatment	56 (47.5)	21 (60.0)	35 (42.2)	0.11
Episodes needing dosage adjustments to avoid overexposure, n (%)	42/56 (73.2)	19/21 (90.5)	22/35 (62.9)	0.031
Duration of linezolid treatment (days), median (IQR)	20 (11–37.5)	16 (11–40)	21 (11–36)	0.96
**Co-treatment, n (%)**				
Amlodipine	16 (13.6)	7 (20.0)	9 (10.8)	0.24
Omeprazole	15 (12.7)	4 (11.4)	11 (13.3)	1.00
Rifampicin	11 (9.3)	5 (14.3)	6 (7.2)	0.30
Amiodarone	2 (1.7)	1 (2.9)	1 (1.2)	0.51
Dexamethasone	2 (1.7)	1 (2.9)	1 (1.2)	0.51
**Other antimicrobials, n (%)**				
Meropenem	26 (22.0)	7 (20.0)	19 (22.9)	0.81
Doripenem	10 (8.5)	1 (2.9)	9 (10.8)	0.28
Piperacillin/tazobactam	15 (12.7)	7 (20.0)	8 (9.6)	0.14
Daptomycin	2 (1.7)	1 (2.9)	1 (1.2)	0.51
Ciprofloxacin	5 (4.2)	0 (0.0)	5 (6.0)	0.32
Levofloxacin	7 (5.9)	0 (0.0)	7 (8.4)	0.10
Micafungin	7 (5.9)	2 (5.7)	5 (6.0)	1.00
Liposomal amphotericin B	4 (3.4)	2 (5.7)	2 (2.4)	0.58
Voriconazole	3 (2.5)	0 (0.0)	3 (3.6)	0.55
**Type of toxicity, n (%)**				
Thrombocytopenia	48 (40.7)	22 (62.9)	26 (31.3)	0.0002
Median time from initiation of therapy to development of thrombocytopenia (*n* = 48), median days (IQR)	12.5 (9.0–15.8)	12.5 (10.8–15)	12.5 (2.8–17.3)	0.56
Severe thrombocytopenia	22 (18.6)	10 (28.6)	12 (14.5)	0.12

*Abbreviations*: *RI* renal impairment, *CL*_*CRC-G*_ creatinine clearance calculated using the Cockcroft-Gault formula, *DIC* disseminated intravascular coagulopathy, *MRSA* methicillin-resistant *Staphylococcus aureus*, *MR-CoNS* methicillin-resistant coagulase-negative staphylococci, *TDM* therapeutic drug monitoring

In the present analyses, a total of 118 episodes contributed 770 linezolid serum *C*_min_ concentrations. Median (IQR) number of instances of TDM were 6 (4–11) in the RI group and 6 (2–8) in the non-RI group. Mean *C*_min_ at steady state for the fixed dose of 600 mg every 12 h in the RI group (25.6 ± 10.4 mg/L) was approximately double that in the non-RI group (14.1 ± 8.8 mg/L, *P* < 0.0001) (Table 1). Patients with episodes in the RI group were older and had lower height, body weight, body mass index, and baseline hemoglobin level. Median duration of linezolid therapy was 16 days in the RI group and 21 days in the non-RI group. Among concomitant medications, amlodipine was the most frequent co-prescribed agent both in total and in the RI group.

The rates of occurrence of thrombocytopenia in the two groups are also reported in Table 1. In total, 48 (40.7%) episodes developed thrombocytopenia and 22 (18.6%) developed severe thrombocytopenia. Thrombocytopenia occurred more frequently among episodes in the RI group (62.9%) than in the non-RI group (31.3%, *P* = 0.0002). Median time from initiation of therapy to development of thrombocytopenia was 12.5 days in both the RI and non-RI groups. In addition, renal impairment was independently associated with an increased risk of thrombocytopenia in uni- and multivariate conditional logistic regression analyses (OR 2.90, 95%CI 1.13–7.44) (Tables 2 and 3). Platelet count at baseline was also found to be independently associated with thrombocytopenia.

**Table 2:** Univariate evaluation of risk factors for development of thrombocytopenia

	Episodes with thrombocytopenia, *n* = 48 (40.7%)	Episodes without thrombocytopenia, *n* = 70 (59.3%)	*P*-value
**Demographics**			
Age (years), median (IQR)	72 (66–77.8)	69 (49.5–78)	0.28
Sex (male/female), (%/%)	32/16 (66.7/33.3)	44/26 (62.9/37.1)	0.70
Height (m), median (IQR)	1.60 (1.51–1.67)	1.63 (1.54–1.68)	0.18
Body weight (kg), median (IQR)	51.5 (45.4–60.2)	60.0 (53.3–65.1)	0.0048
Body mass index (kg/m^2^), median (IQR)	21.1 (19.1–23.3)	22.5 (20.7–25.3)	0.0082
**Laboratory, median (IQR)**			
Serum creatinine (mg/dL)	0.80 (0.52–1.26)	0.60 (0.50–0.83)	0.040
CL_CRC-G_ ≤ 60 mL/min	22 (45.8)	13 (18.6)	0.0020
Total bilirubin (mg/dL)	0.4 (0.3–0.6)	0.5 (0.3–0.73)	0.43
**Baseline haematological parameters, median (IQR)**			
Hemoglobin concentration (g/dL), median (IQR)	9.3 (8.4–10.5)	10.0 (8.7–11.8)	0.033
Platelet count (×10^3^/μL), median (IQR)	205 (143.5–254.5)	303.5 (195–382.5)	< 0.0001
Low platelet count at baseline < 150 × 10^3^/μL, n (%)	13 (27.1)	10 (14.3)	0.101
Episodes with platelet transfusion during therapy, n (%)	6 (12.5)	2 (2.9)	0.061
Episodes with DIC, n (%)	9 (18.8)	6 (8.6)	0.16
**Main reason for linezolid**			
**Type of infection, n (%)**			
Skin and soft tissue infections, and surgical site infections	17 (35.4)	30 (42.9)	0.45
Bacteraemia	19 (39.6)	17 (24.3)	0.103
Bone and joint infections	13 (27.1)	18 (25.7)	1.00
Respiratory tract infections	9 (18.8)	17 (24.3)	0.51
Intra-abdominal infections	3 (6.3)	5 (7.1)	1.00
Mediastinitis	4 (8.3)	3 (4.3)	0.44
Central nervous system infections	0 (0.0)	5 (7.1)	0.079
Endocarditis	2 (4.2)	2 (2.9)	1.00
Urinary tract infections	2 (4.2)	2 (2.9)	1.00
Unknown	3 (6.3)	3 (4.3)	0.69
**Linezolid dosage and exposure**			
Empirical/target therapy, n/n (%/%)	6/48 (12.5/87.5)	11/59 (15.7/84.3)	0.79
Mean *C*_min_ of fixed doses in steady state (mg/L), mean ± SD	20.6 ± 10.8	15.3 ± 9.8	0.0023
Duration of linezolid treatment (days), median (IQR)	21 (12–42.8)	19.5 (10.8–34.3)	0.29

*Abbreviations*: *CL*_*CRC-G*_ creatinine clearance calculated using the Cockcroft-Gault formula, *DIC* disseminated intravascular coagulopathy

**Table 3:** Multivariate conditional logistic regression analysis of variables associated with occurrence of thrombocytopenia

	OR (95%CI)	*P*-value
Male	1.25 (0.52–3.01)	0.62
Body mass index (kg/m^2^) (per 1-kg/m^2^ increment)	0.93 (0.72–1.08)	0.25
CL_CRC-G_ ≤ 60 mL/min	2.90 (1.13–7.44)	0.027
Hemoglobin concentration (g/dL) (per 1-g/dL increment)	0.89 (0.72–1.08)	0.23
Platelet count (×10^3^/μL) (per 1.0 × 10^3^/μL increment)	0.993 (0.989–0.997)	0.0002
Bacteraemia	1.44 (0.51–4.01)	0.49
Duration of linezolid treatment (days) (per 1-day increment)	1.010 (0.989–1.031)	0.36

R^2^ = 0.189

*Abbreviations*: *CL*_*CRC-G*_ creatinine clearance calculated using the Cockcroft-Gault formula

Because many other confounding factors could affect linezolid overexposure, effects were further analyzed by multivariate linear regression using *C*_min_ collected after the fixed dose of 600 mg every 12 h (Table 4). Renal impairment and total body weight were independent predictors of higher *C*_min_ at the standard dose (R^2^ = 0.30). However, linezolid *C*_min_ correlated linearly but weakly with CL_CRC-G_ (adjusted R^2^ = 0.234, *P* < 0.0001) (Supplemental Fig. 1) and total body weight (0.142, *P* < 0.0001). Similarly, linezolid *C*_min_ correlated only weakly with other factors including age (adjusted R^2^ = 0.185, *P* < 0.0001) and body mass index (0.047, *P* = 0.013). Inter-episode coefficients of variation for linezolid *C*_min_ were 40.6% in the RI group and 61.7% in the non-RI group. Therefore, it should not be overlooked that renal function seems to partially explain the wide interindividual variability in *C*_min_ observed in this study population.

**Table 4:** Uni- and multivariate linear regression analysis of variables associated with linezolid *C*_min_ at standard dose of 600 mg every 12 h

Variables	Univariate analysis		Multivariate analysis^a^	
	Unstandardized β coefficient (95%CI)	*P*-value	Unstandardized β coefficient (95%CI)	*P*-value
Male	−2.81 (−6.95 to 1.34)	0.18		
Age (years) (per 1-year increment)	0.285 (0.173 to 0.396)	< 0.0001		
Height (m) (per 1-m increment)	−33.71 (−51.35 to −16.07)	0.0003		
Body weight (kg) (per 1-kg increment)	−0.294 (−0.427 to −0.160)	< 0.0001	−0.208 (−0.335 to 0.081)	0.0016
CL_CRC-G_ ≤ 60 mL/min	11.37 (7.397 to 15.345)	< 0.0001	4.777 (2.793 to 6.760)	< 0.0001
Total bilirubin > 1.2 mg/dL	1.111(−2.960–5.199)	0.59		
**Co-treatment**				
Omeprazole	−1.097 (−7.273 to 5.079)	0.73		
Amiodarone	4.676 (−1.243 to 10.595)	0.12		
Amlodipine	1.037 (−13.885 to 15.960)	0.89		
Rifampicin	2.236 (−5.028 to 9.501)	0.54		
Dexamethasone	0.426 (−14.450 to 15.350)	0.96		

^a^R^2^ = 0.301

*Abbreviation*: *CL*_*CRC-G*_ creatinine clearance calculated using the Cockcroft-Gault formula

### Usefulness of TDM

**Fig. 2:**
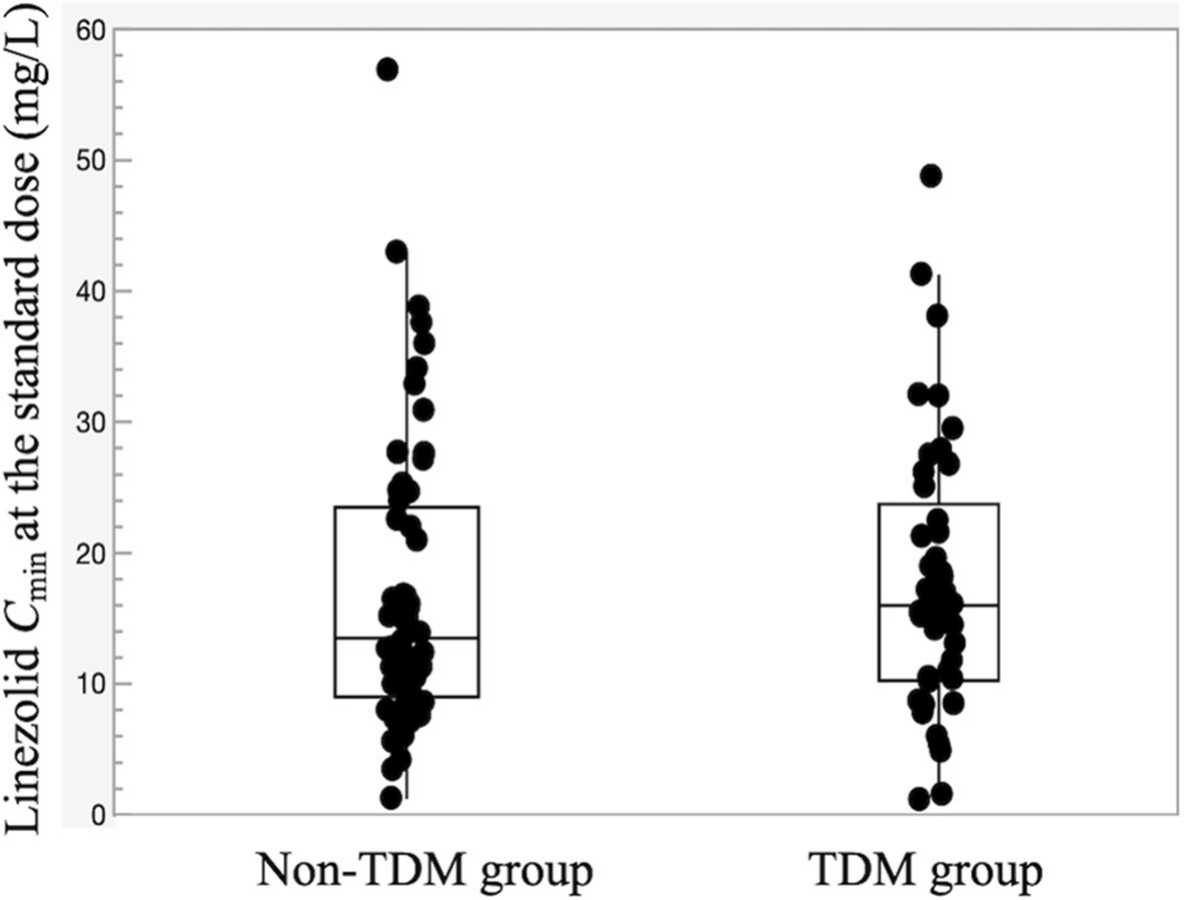
Boxplots of *C*_min_ at the standard dose in the TDM group and non-TDM group. For each boxplot, the horizontal line across the box within each box represents the median, each box represents the range between the 25th and 75th percentiles, the two whiskers represent the minimum and maximum values that are within 1.5 × IQR, and points beyond the whiskers represent outliers. Closed circles represent *C*_min_ at the standard dose. Abbreviations: *C*_min_, trough concentration; TDM, therapeutic drug monitoring

In the analysis of the usefulness of TDM, the TDM group comprised 56 episodes from 52 patients and the non-TDM group comprised 62 episodes from 61 patients. Episodes in the two groups were further separated by the presence or absence of renal impairment. The distributions of *C*_min_ at the standard dose for both groups in the TDM and non-TDM groups were represented in Fig. 2. When assessing these episodes in terms of length of treatment and clinical outcome (Table 5), the duration of linezolid treatment was significantly longer in the TDM group than in the non-TDM group. No significant differences were seen among the TDM and non-TDM groups in failure rate due to persistence of infection. In addition, although thrombocytopenia occurred more frequently among episodes in the TDM group, failure rate due to toxicity and/or persistence of infection tended to be higher in the non-TDM group, but the difference did not reach statistical significance (*P* = 0.052). Failure rates did not differ significantly between the two groups in the non-RI group. On the other hand, although there was no significant difference with respect to the general characteristics, baseline hematological parameters and concomitant drug treatments, failure in general, and due to hematological toxicity were significantly lower in the TDM group of the RI group (Table 5).

**Table 5:** Clinical outcome and length of treatment in TDM and non-TDM groups, further separated by presence or absence of renal impairment (RI or non-RI groups)

**Total**	TDM group, *n* = 56 (47.5%)	Non-TDM group, *n* = 62 (52.5%)	*P*-value
Recovery, n (%)	38 (67.9)	35 (56.5)	0.26
Duration of linezolid treatment (days), median (IQR)	30 (19.5–45)	12 (9–21.3)	< 0.0001
Failure, n (%)	14 (25.0)	27 (43.6)	0.052
Failure due to persistence of infection, n (%)	6 (10.7)	2 (3.2)	0.15
Failure due to hematological toxicity, n (%)	10 (17.9)	18 (29.0)	0.20
Failure due to other toxicity, n (%)	3 (5.4)	8 (12.9)	0.21
Thirty-day reinfection, n (%)	5 (8.9)	4 (6.5)	0.73
Thrombocytopenia	32 (66.7)	16 (33.3)	0.0007
**RI group**	TDM group, *n* = 21, (60.0%)	Non-TDM group, *n* = 14, (40.0%)	*P*-value
Recovery, n (%)	15 (71.4)	5 (35.7)	0.080
Duration of linezolid treatment (days), median (IQR)	34 (20–46)	11.5 (8.8–13.3)	< 0.0001
Failure, n (%)	3 (14.3)	9 (64.3)	0.0038
Failure due to persistence of infection, n (%)	1 (4.8)	0 (0.0)	1.00
Failure due to hematological toxicity, n (%)	2 (9.5)	8 (57.1)	0.0056
Failure due to other toxicity, n (%)	0 (0.0)	2 (14.3)	0.15
Thirty-day reinfection, n (%)	3 (14.3)	1 (7.1)	0.64
Thrombocytopenia	17 (81.0)	5 (35.7)	0.012
**Non-RI group**	TDM group, *n* = 35 (42.2%)	Non-TDM group, *n* = 48 (57.8%)	*P*-value
Recovery, n (%)	23 (65.7)	30 (62.5)	0.82
Duration of linezolid treatment (days), median (IQR)	29 (19–45)	13.5 (9–22.8)	< 0.0001
Failure, n (%)	18 (37.5)	11 (31.4)	0.64
Failure due to persistence of infection, n (%)	2 (4.2)	5 (14.3)	0.13
Failure due to hematological toxicity, n (%)	10 (20.8)	8 (22.9)	1.00
Failure due to other toxicity, n (%)	6 (12.5)	3 (8.6)	0.73
Thirty-day reinfection, n (%)	3 (6.3)	2 (5.7)	1.00
Thrombocytopenia	15 (42.9)	11 (22.9)	0.060

*Abbreviations*: *TDM* therapeutic drug monitoring, *RI* renal impairment

**Fig. 3:**
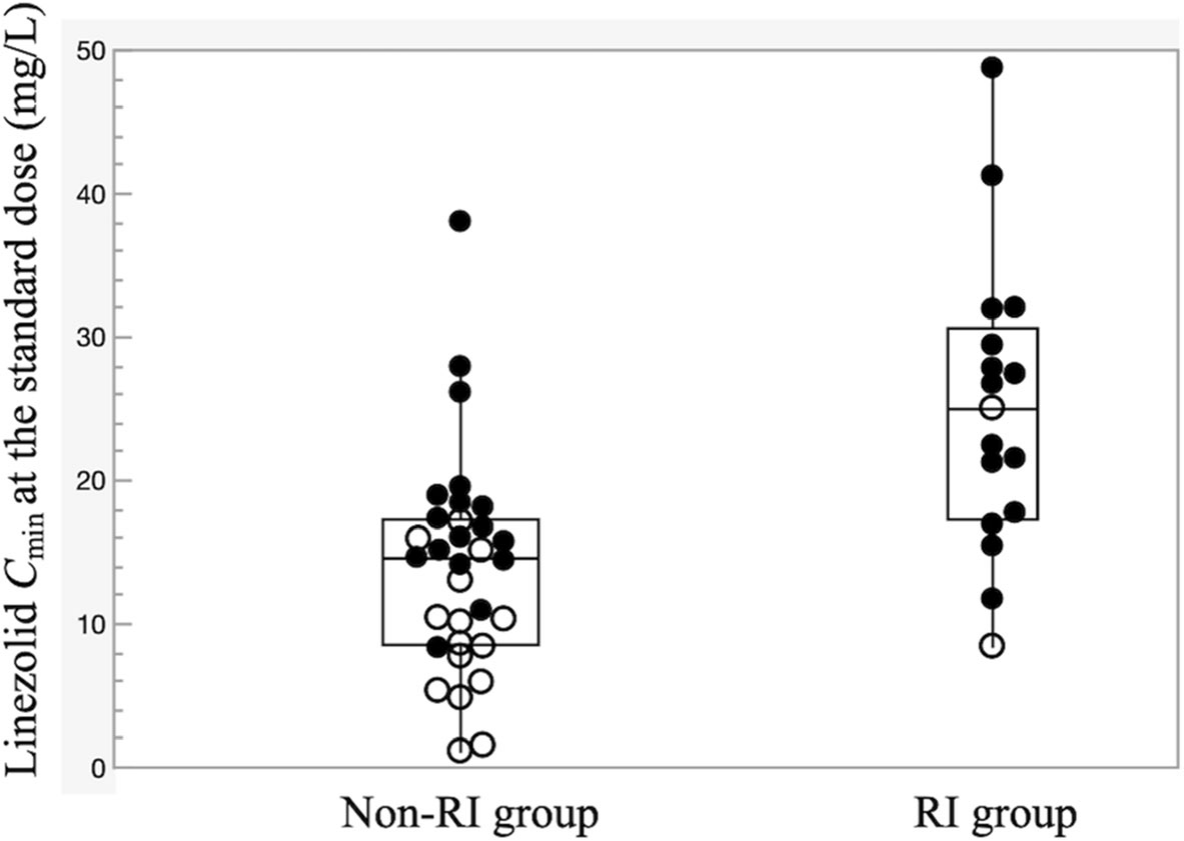
Boxplots of *C*_min_ at the standard dose in the RI and non-RI groups among the episodes in the TDM group. For each boxplot, the horizontal line across the box within each box represents the median, each box represents the range between the 25th and 75th percentiles, the two whiskers represent the minimum and maximum values that are within 1.5 × IQR, and points beyond the whiskers represent outliers. Closed circles represent *C*_min_ values of the episodes in which dose adjustment was performed, and open circles represent *C*_min_ values of the episodes in which dose adjustment was not performed. Abbreviations: *C*_min_, trough concentration; RI, renal impairment, TDM, therapeutic drug monitoring

**Fig. 4:**
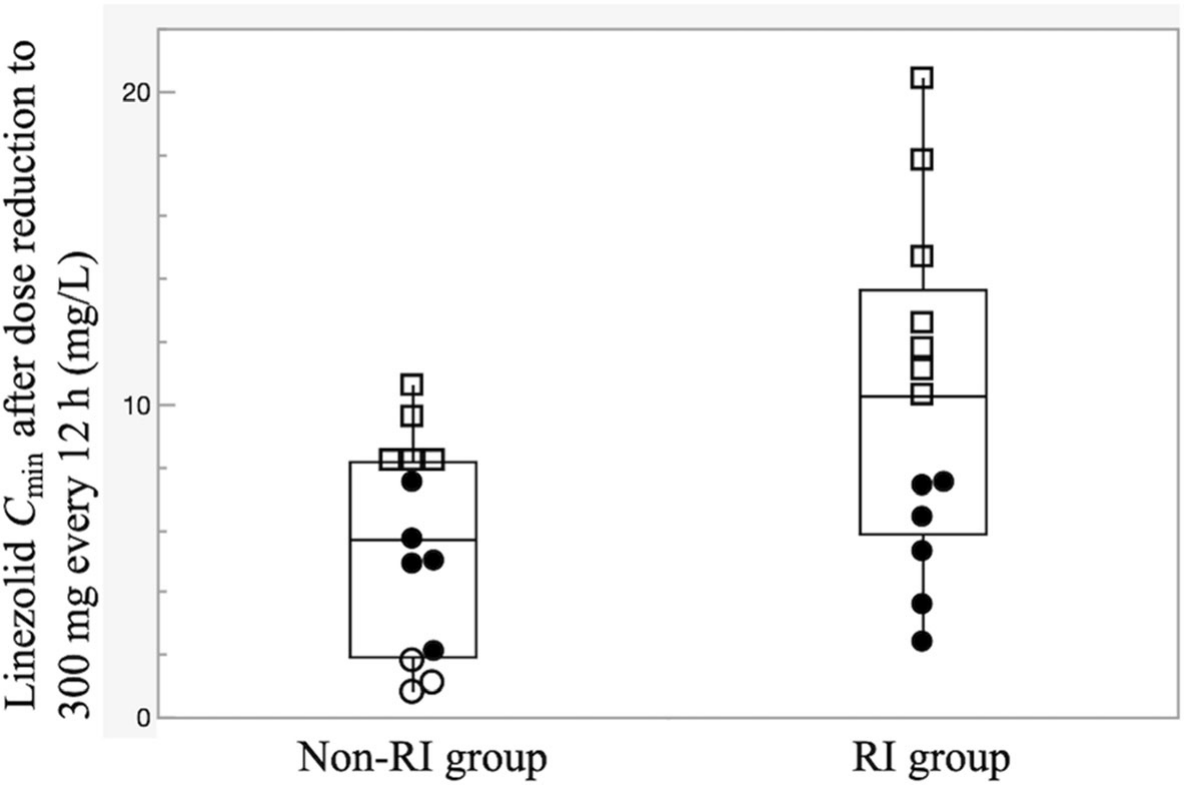
Boxplots of *C*_min_ after dose reduction to 300 mg every 12 h in the RI and non-RI groups. For each boxplot, the horizontal line across the box within each box represents the median, each box represents the range between the 25th and 75th percentiles, the two whiskers represent the minimum and maximum values that are within 1.5 × IQR, and points beyond the whiskers represent outliers. Open circles represent *C*_min_ < 2.0 mg/L, closed circles represent *C*_min_ within the desired range of 2–8 mg/L, and open square represent *C*_min_ values of overexposure (> 8 mg/L). Abbreviations: *C*_min_, trough concentration; RI, renal impairment

In the TDM group, dosage adjustments over time to avoid potential linezolid overexposure were needed in 90.5% of episodes in the RI group compared to only 62.9% of episodes in the non-RI group (*P* = 0.031) (Fig. 3). TDM-guided dosage reductions allowed recovery from thrombocytopenia and prosecution of therapy until the planned end of treatment with good outcome in 12 (37.5%) of 32 episodes experiencing thrombocytopenia in the TDM group. Of the episodes needing dose reduction in the TDM group, all those episodes administered a reduced dose of 300 mg every 12 h in the RI group and in which steady-state *C*_min_ of the reduced dose could be measured (*n* = 13) showed *C*_min_ ≥ 2.0 mg/L, with no episode experiencing linezolid underexposure (Fig. 4). On the other hand, in the non-RI group, 62.9% of episodes in patients were needed for dose reduction, but 23.1% (3/13) of these episodes were under exposure (< 2 mg/L) when administered reduced dose of 300 mg every 12 h (Fig. 4). Mean *C*_min_ at the time of dose reduction to 300 mg every 12 h was significantly higher (10.1 ± 5.4 mg/L) than that in the non-RI group (n = 13, 5.7 ± 3.4 mg/L, *P* = 0.038). Based on these results, a reduced dose of 300 mg every 12 h may be recommended as a maintenance dose in patients with renal impairment rather than patients with preserved renal function. However, despite using a reduced linezolid dose of 300 mg every 12 h, achieving linezolid *C*_min_ within the optimal range was seen in only 46.2% (6/13) of episodes in the RI group and 38.5% (5/13) in the non-RI group (Fig. 4). TDM-based further reduction to 300 mg once daily was needed in 23.8% (5/21) of episodes in the RI group.

### Initial and maintenance dosing strategy

In an additional analysis of 53 episodes in which *C*_min_ was measured within 48 h of starting administration of a fixed 600 mg every 12 h, linezolid *C*_min_ of first measurement (first *C*_min_) at 12, 24, 36, and 48 h after start administration were significantly higher in the RI group than in the non-RI group. The minimal first *C*_min_ in the RI group was the *C*_min_ of 2.9 mg/L at 24 h after start administration and all these episodes in the RI group were above the minimum value of optimal range (> 2 mg/L) even within 48 h after starting administration (Table 6 and Supplemental Fig. 2). On the other hand, some first *C*_min_ of the episodes in the non-RI group were underexposure (Table 6 and Supplemental Fig. 2).

**Table 6:** Linezolid *C*_min_ of the first measurement (first *C*_min_) at 12, 24, 36, or 48 h after starting administration of fixed 600 mg every 12 h and ratio of first *C*_min_ to mean *C*_min_ under steady state in the RI group and non-RI group

Time after starting administration of fixed 600 mg every 12 h (h)	Linezolid *C*_min_ of first measurement (first *C*_min_), mean ± SD (range)					Ratio of first *C*_min_ to mean *C*_min_ under steady state, median (IQR)				
	RI group	n	Non-RI group	n	*P*-value	RI group	n	Non-RI group	n	*P*-value
12	8.9 ± 0.4 (8.6–9.4)	3	6.2 ± 3.7 (0.2–14.0)	17	0.090	52.0 (24.3–75.6)	3	52.0 (26.4–80.7)	17	0.96
24	12.3 ± 8.8 (2.9–24.3)	7	8.3 ± 3.6 (4.8–14.2)	5	0.75	81.8 (64.4–118.3)	7	58.2 (27.3–64.4)	5	0.051
36	18.8 ± 3.6 (16.2–21.3)	2	9.6 ± 7.8 (0.5–23.3)	7	0.19	67.6 (41.7–102.0)	2	61.3 (45.1–77.5)	7	0.88
48	25.3 ± 9.6 (15.8–36.1)	4	8.7 ± 4.8 (1.4–14.7)	8	0.0085	62.3 (45.8–79.1)	4	79.1 (55.0–86.8)	8	0.44
Total	15.7 ± 9.5 (2.9–36.1)	16	7.7 ± 4.9 (0.2–23.3)	37	0.0019	58.9 (37.8–89.1)	16	60.5 (38.7–79.9)	37	0.71

*Abbreviations*: *C*_*min*_ trough concentration, *first C*_*min*_ *C*_min_ of first measurement, *RI* renal impairment

In addition to the observational real-world data from clinical practice, we performed the linezolid dosing simulation of the hypothetical patient with mild renal impairment (60 years old; total body weight, 70 kg; CL_CRC-G_, 60 mL/min), using recently accepted simulation software “Pycsim” based on population pharmacokinetic and pharmacodynamic model [15, 20]. When linezolid was initially administered at a dose of 600 mg via hypothetical intravenous drip infusion for 60 min at 12-h intervals for 2 days, and thereafter reduced dose of 300 mg via hypothetical intravenous drip infusion for 60 min every 12 h, the simulated *C*_min_ at the 48 h after start administration and steady-state *C*_min_ at the reduced dose of 300 mg every 12 h were 9.8 and 5.2 mg/L, respectively. These data suggested that initial administration of a fixed dose for 2 days may be sufficient to rapidly reach an effective therapeutic concentration and empirical dose reduction to 300 mg every 12 h under TDM control may provide the best balance of safety and efficacy, achieving therapeutic concentrations (2–8 mg/L) in patients with CL_CRC-G_ ≤ 60 mL/min.

## Discussion

Several previous studies have shown that patients with renal impairment more frequently experienced thrombocytopenia during fixed dose treatment [9, 21, 22]. Similarly, in our study, we demonstrate a 3 times greater risk of thrombocytopenia with CL_CRC-G_ ≤ 60 mL/min. These high frequencies of thrombocytopenia in patients with impaired renal function may be due to increased linezolid concentrations and the absence of specific indications on dose adjustments according to renal function.

Indeed, the present study found that the mean *C*_min_ of episodes with renal impairment was approximately double (25.6 ± 10.4 mg/L) that of episodes without renal impairment (14.1 ± 8.8 mg/L, *p* < 0.0001). Renal impairment was thus an independent predictor of higher *C*_min_ of the fixed dose, consistent with previous reports [4, 12].

However, many other covariates, including liver dysfunction, have been reported to affect the pharmacokinetics of linezolid [6, 23, 24]; therefore, a population pharmacokinetics approach would be preferred over the simplistic assessment of trough concentrations to evaluate the influence of renal impairment on linezolid clearance. Although we did not perform population pharmacokinetics analysis, in our previous analysis of linezolid population pharmacokinetics in 81 patients of similar background, about 50% of elimination was found to be explained by renal clearance [15]. Similarity, several population pharmacokinetics studies using data obtained from clinical practice have also consistently demonstrated renal function to be one of the most important predictor of linezolid clearance [6, 11, 18, 25] and the results of the present study reconfirmed the necessity of effective linezolid dose adjustment for renal impairment patients.

Previous studies have therefore suggested that a reduced dose of 300 mg every 12 h be recommended for patients with CL_CR_ < 30 mL/min or eGFR < 60 mL/min/1.73m^2^, based on Monte Carlo simulations for sufficient efficacy and safety [9, 18]. However, to the best of our knowledge, no previous studies have supported this recommendation with actual measurement data from clinical practice. Furthermore, no studies appear to have considered the initial and maintenance dosing regimens separately.

Notably, in the present analyses, we found that an empirical dose reduction to 300 mg every 12 h under TDM control may provide the best balance of safety and efficacy in Japanese patients with renal impairment, with no patients exposed to sub-therapeutic linezolid concentrations after dose reduction to 300 mg every 12 h (Table 4). Further, we suggested that the initial fixed dose administration for 2 days was enough to rapidly reach an effective therapeutic concentration in the present additional analyses based on the actual measurement data (Table 6 and Supplemental Fig. 2) and the simulation data of linezolid concentrations using recently accepted simulation software (Supplemental Fig. 3) [15, 20].

Despite using a reduced linezolid dose of 300 mg every 12 h, Crass et al. demonstrated the simulated probability of achieving linezolid *C*_min_ within the therapeutic range of 2–8 mg/L was only approximately 65% in simulated patients with eGFR < 60 mL/min/1.73m^2^. Similarly, in the present study, achieving linezolid *C*_min_ within the therapeutic range was seen in only 46.2% of episodes in the RI group even after dose reduction to 300 mg every 12 h (Fig. 4). Furthermore, TDM-based further reduction to 300 mg once daily was needed in 23.8% (5/21) of episodes in the RI group. On the other hand, in the non-RI group, 63% of episodes administered the fixed dose were also needed for dose reduction and despite using a reduced linezolid dose of 300 mg every 12 h, achieving linezolid *C*_min_ within the therapeutic range was seen in only 38.5% (5/13) in the non-RI group. All these observed results may be due to the large unexplained interindividual variation on clearance.

With regard to linezolid clearance, CL_CR_ was identified as the only covariate that significantly explained between subject variation [6], whereas variability due to other unknown factors still remained (the interindividual variability in clearance = 31.3%) in our previous study [11] and was nearly equivalent to previously reported values (30.5% [6] and 35.2% [18]). Renal dose adjustments alone are thus unlikely to ensure adequate safety and efficacy of linezolid with prolonged therapy.

The use of TDM for patients who require prolonged linezolid treatment is thus essential to any intervention evaluating empirical dose reduction in patients with renal impairment. Also, even in patients with preserved renal function, although empirical dose reduction may not be recommended because of the presence of some episodes with underexposure, TDM and dose reduction under TDM control may also be needed to avoid overexposure and treatment failure. Pea et al. found that TDM-guided dose modification facilitates resolution of thrombocytopenia and safe continuation of therapy in one-third of patients who developed toxicity on standard empirical doses [4]. Similarly, we found that TDM-guided dosage adjustments to maintain the linezolid *C*_min_ range of 2–8 mg/L allowed recovery from thrombocytopenia and prosecution of therapy until the planned end of treatment, with good outcomes in 12 (37.5%) of 32 episodes experiencing thrombocytopenia among both patients with renal impairment and preserved renal function.

This study showed limitations inherent to the retrospective design and potential for confounding clinical conditions that cannot be excluded. We used multivariable models to control for confounding patient and clinical factors, but the potential for residual confounding remains. Furthermore, reliance on nominal times of administration and sample collection based on standards of care may have influenced the observed interindividual variability and led to misspecification due to deviations from the sampling protocol in clinical practice. However, our results are consistent with previously published studies, which increases confidence in the results.

## Conclusions

In conclusion, our findings indicate that TDM-guided dose adjustment to maintain the linezolid *C*_min_ range of 2–8 mg/L may be beneficial in preventing treatment failure and in recovering from exposure-dependent thrombocytopenia. Initial fixed-dose administration for 2 days may be enough to rapidly reach an effective therapeutic concentration and empirical dose reduction to 300 mg every 12 h under TDM control may provide the best balance of safety and efficacy in Japanese patients with CL_CRC-G_ ≤ 60 mL/min. Further clinical studies involving a large number of patients are necessary to validate our results.

## Supplementary Information

**Additional file 1: Supplemental Figure 1.** Relationship between linezolid *C*_min_ of the fixed dose of 600 mg every 12 h and creatinine clearance as estimated using the Cockcroft-Gault formula (CL_CRC-G_). Abbreviations: *C*_min_, trough concentration; CL_CRC-G_, creatinine clearance calculated using the Cockcroft-Gault formula.

**Additional file 2: Supplemental Figure 2.** Dot plots represent the distribution of linezolid *C*_min_ of the first measurement (first *C*_min_) at 12, 24, 36, or 48 h after starting administration of fixed 600 mg every 12 h in the RI group (A) and the non-RI group (B). Open circles represent *C*_min_ < 2.0 mg/L, closed circles represent *C*_min_ within the desired range of 2–8 mg/L, and open square represent *C*_min_ values of overexposure (> 8 mg/L). Abbreviations: *C*_min_, trough concentration; fist *C*_min,_ *C*_min_ of first measurement; RI, renal impairment.

**Additional file 3: Supplemental Figure 3.** Simulation of linezolid concentrations using Pycsim software. Shown are screenshots of the application running in the browser-window. This capture is the result of simulation performed after input of the dosing records based on hypothetical patients with mild renal impairment. The dosing records were inputted as initial administration at a dose of 600 mg via hypothetical intravenous drip infusion for 60 min at 12-h intervals for 2 days, and thereafter reduced dose administration of 300 mg via hypothetical intravenous drip infusion for 60 min every 12 h. The final output is a file consisting of both parts; the left column represents population prediction with pharmacokinetic parameters, the right column represents the simulation curve of total and unbound concentration (black lines: population prediction).

## Data Availability

All data generated or analysed during this study are included in this published article.

## Abbreviations

MRSA: Methicillin-resistant *Staphylococcus aureus*
MR-CoNS: Methicillin-resistant coagulase-negative staphylococci
*C*_min_: Trough concentration
CL: Clearance
TDM: Therapeutic drug monitoring
CL_CR_: Creatinine clearance
eGFR: Estimated glomerular filtration rate
CL_CRC-G_: CL_CR_ calculated using the Cockcroft-Gault formula
ID: Infectious disease
first *C*_min_: *C*_min_ of first measurement
