# Supplementary figures and images for: Proposal of initial and maintenance dosing regimens with linezolid for renal impairment patients
Source: BMC Pharmacol Toxicol. 2021 Mar 4;22:13. doi: 10.1186/s40360-021-00479-w (PMC7934392; doi:10.1186/s40360-021-00479-w)

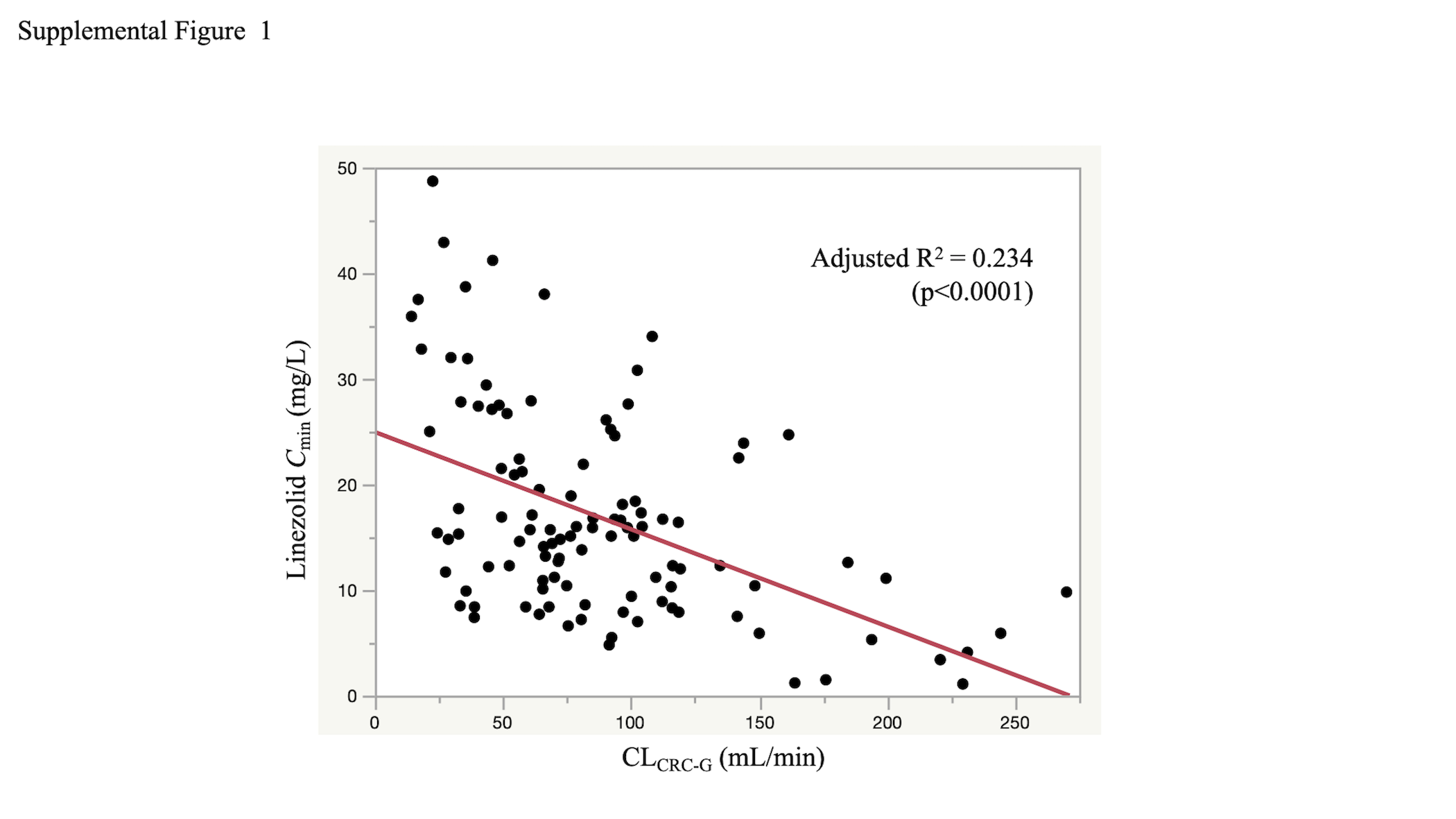

Supplement: Supplementary file 1 — Additional file 1: Supplemental Figure 1. Relationship between linezolid Cmin of the fixed dose of 600 mg every 12 h and creatinine clearance as estimated using the Cockcroft-Gault formula (CLCRC-G). Abbreviations: Cmin, trough concentration; CLCRC-G, creatinine clearance calculated using the Cockcroft-Gault formula. [file 40360_2021_479_MOESM1_ESM.tiff]

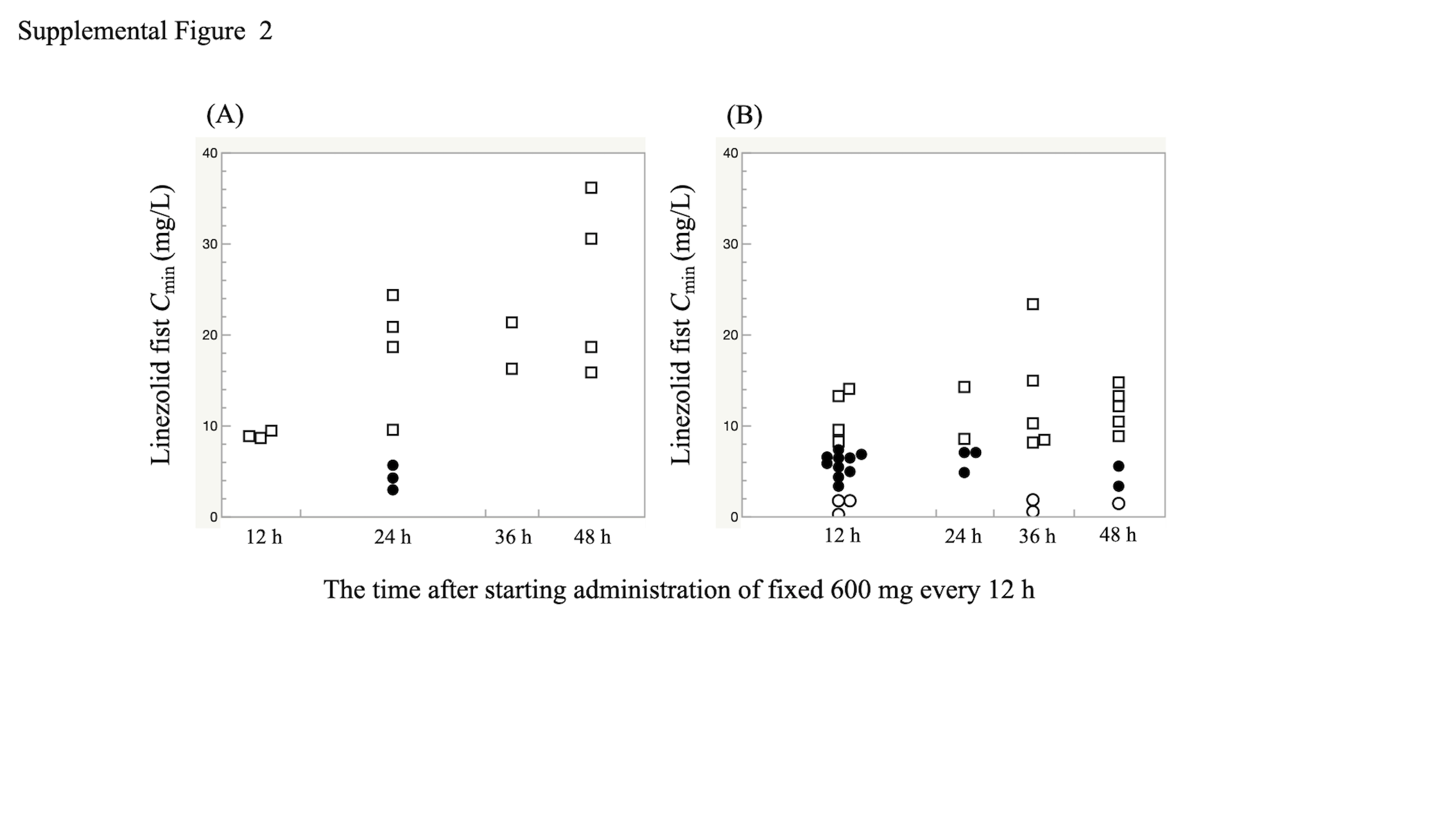

Supplement: Supplementary file 2 — Additional file 2: Supplemental Figure 2. Dot plots represent the distribution of linezolid Cmin of the first measurement (first Cmin) at 12, 24, 36, or 48 h after starting administration of fixed 600 mg every 12 h in the RI group (A) and the non-RI group (B). Open circles represent Cmin < 2.0 mg/L, closed circles represent Cmin within the desired range of 2–8 mg/L, and open square represent Cmin values of overexposure (> 8 mg/L). Abbreviations: Cmin, trough concentration; fist Cmin, Cmin of first measurement; RI, renal impairment. [file 40360_2021_479_MOESM2_ESM.tiff]

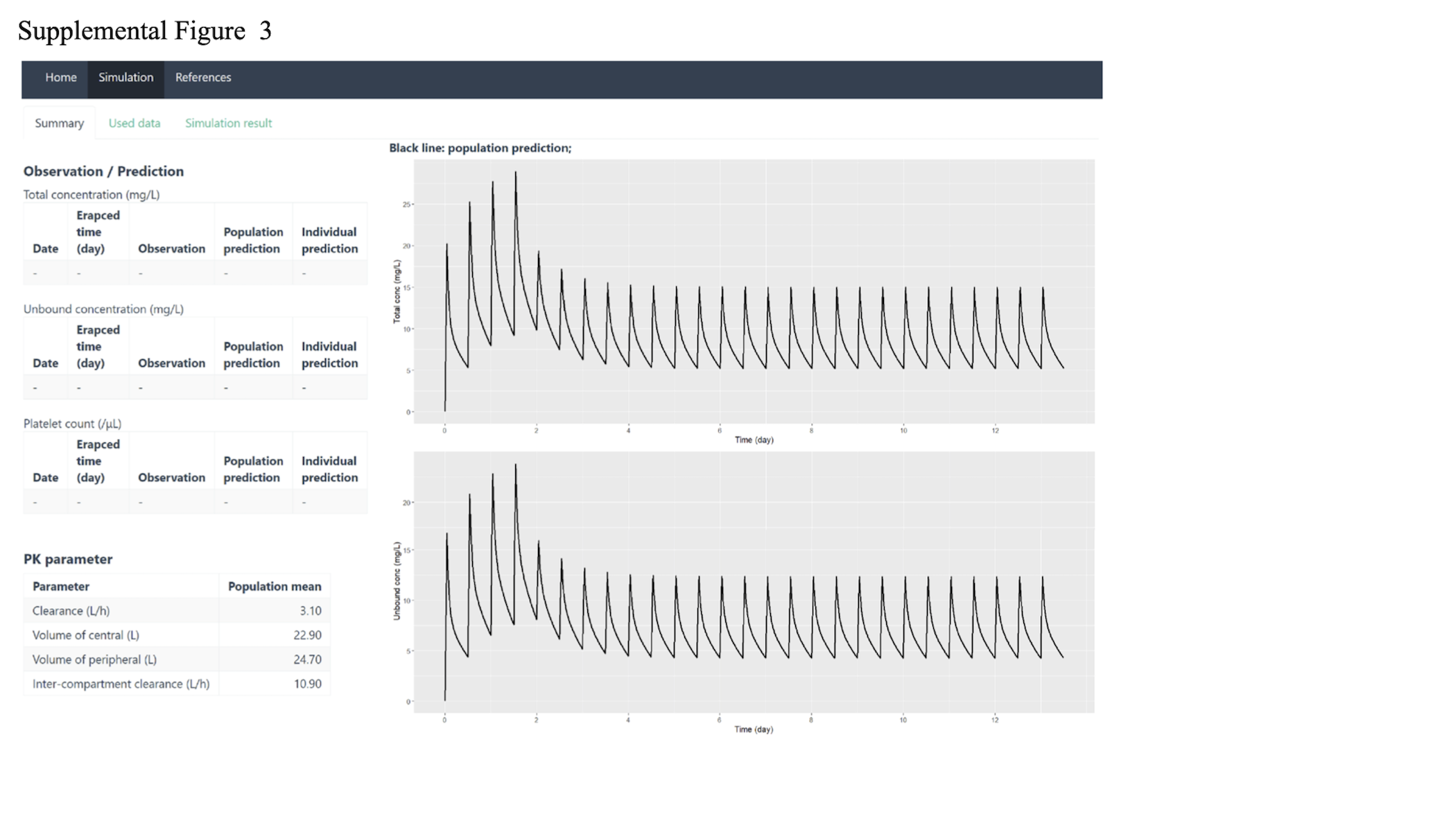

Supplement: Supplementary file 3 — Additional file 3: Supplemental Figure 3. Simulation of linezolid concentrations using Pycsim software. Shown are screenshots of the application running in the browser-window. This capture is the result of simulation performed after input of the dosing records based on hypothetical patients with mild renal impairment. The dosing records were inputted as initial administration at a dose of 600 mg via hypothetical intravenous drip infusion for 60 min at 12-h intervals for 2 days, and thereafter reduced dose administration of 300 mg via hypothetical intravenous drip infusion for 60 min every 12 h. The final output is a file consisting of both parts; the left column represents population prediction with pharmacokinetic parameters, the right column represents the simulation curve of total and unbound concentration (black lines: population prediction). [file 40360_2021_479_MOESM3_ESM.tiff]
